# CRISPR/Cas9-Mediated Knockout of the Lycopene ε-Cyclase for Efficient Astaxanthin Production in the Green Microalga *Chlamydomonas reinhardtii*

**DOI:** 10.3390/plants13101393

**Published:** 2024-05-17

**Authors:** Jacob Sebastian Kneip, Niklas Kniepkamp, Junhwan Jang, Maria Grazia Mortaro, EonSeon Jin, Olaf Kruse, Thomas Baier

**Affiliations:** 1 Algae Biotechnology and Bioenergy, Faculty of Biology, Center for Biotechnology (CeBiTec), Bielefeld University, 33615 Bielefeld, Germany; 2 Department of Life Science, Research Institute for Natural Sciences, Hanyang University, Seoul 04763, Republic of Korea

**Keywords:** *Chlamydomonas reinhardtii*, genome editing, astaxanthin production, microalgal carotenoids, metabolic engineering, lycopene ß-cyclase, lycopene ε-cyclase

## Abstract

Carotenoids are valuable pigments naturally occurring in all photosynthetic plants and microalgae as well as in selected fungi, bacteria, and archaea. Green microalgae developed a complex carotenoid profile suitable for efficient light harvesting and light protection and harbor great capacity for carotenoid production through the substantial power of the endogenous 2-C-methyl-D-erythritol 4-phosphate (MEP) pathway. Previous works established successful genome editing and induced significant changes in the cellular carotenoid content in *Chlamydomonas reinhardtii*. This study employs a tailored carotenoid pathway for engineered bioproduction of the valuable ketocarotenoid astaxanthin. Functional knockout of lycopene ε-cyclase (LCYE) and non-homologous end joining (NHEJ)-based integration of donor DNA at the target site inhibit the accumulation of α-carotene and consequently lutein and loroxanthin, abundant carotenoids in *C. reinhardtii* without changes in cellular fitness. PCR-based screening indicated that 4 of 96 regenerated candidate lines carried (partial) integrations of donor DNA and increased ß-carotene as well as derived carotenoid contents. Iterative overexpression of *Cr*BKT, *Pa*crtB, and *Cr*CHYB resulted in a 2.3-fold increase in astaxanthin accumulation in mutant ΔLCYE#3 (1.8 mg/L) compared to the parental strain UVM4, which demonstrates the potential of genome editing for the design of a green cell factory for astaxanthin bioproduction.

## 1. Introduction

The green microalga *Chlamydomonas reinhardtii* is an established model organism for basic research in photosynthesis, cilia biogenesis, and phototaxis and is a promising next-generation platform organism for sustainable bioproduction. It combines the ability for phototrophic cultivation and simple genetic manipulation for the engineered synthesis of numerous valuable products ranging from platform chemicals [1,2,3] to pharmaceutical proteins [4] and high-value secondary metabolites [5,6,7,8]. Cultivation of green microalgae in simple mineral salt solutions allows the direct conversion of waste carbon (e.g., CO_2_ or acetate) into industrial-relevant products and offers great potential to establish a resource-efficient, decentralized bioeconomy [8].

*C. reinhardtii* natively harbors a complex pigment profile composed of chlorophylls and carotenoids, each with individual value for biotechnological application. They mediate a dynamically regulated capacity for light harvesting, while excess light is effectively quenched by photoprotection mechanisms. Rapid carotenoid biosynthesis is fueled by the metabolic power of the methyl-D-erythritol phosphate (MEP) pathway located in the chloroplast. It provides sufficient amounts of isopentenyl pyrophosphate (IPP) and dimethylallyl pyrophosphate (DMAPP) as building blocks for phytoene, the shared precursor of all endogenous carotenoids. The enzymes phytoene desaturase (PDS) and ζ-carotene desaturase (ZDS) catalyze the conversion into bright-red-colored lycopene followed by pathway branching into α- and ß-carotenes with ε/β- or ß/β-ionone-type end groups, respectively, by the activity of two competing lycopene cyclases (LCYB/E). In *C. reinhardtii,* α-carotene is hydroxylated by the activity of P450 ε/ß-ring hydroxylases (CYP97A and CYP97C) [9] at both C3 positions for the synthesis of lutein, a common, yellow–red colored xanthophyll found in all green plants and their progenitors. The native cellular abundance in *C. reinhardtii* varies depending on the present light intensity [10], is reported to range between 1.77 and 4.13 mg/g [11,12,13], and was engineered to 8.9 mg/g [12] by the overexpression of *Xanthophyllomyces dendrorhous* phytoene-β-carotene synthase (*crtYB*). An alternative strategy, by the overexpression of *C. reinhardtii* LCYE, resulted in a 2.6-fold increased lutein production (to 0.48 mg/L) [14]. Lutein can be further hydroxylated at the C9 position to form loroxanthin by a yet unknown enzyme [15], and the lutein–loroxanthin cycle has a potential function in non-photochemical quenching (NPQ) [10,16,17,18].

The ß-carotene branch in *C. reinhardtii* continues by enzymatic hydroxylation via ß-carotene hydroxylase (CHYB) and results in the formation of zeaxanthin, a frequent yellow-colored xanthophyll and the start point of the photoprotective xanthophyll cycle. Zeaxanthin is subject to epoxidation from reactive oxygen species (ROS) in excess light or via zeaxanthin epoxidase (ZEP) activity to form antheraxanthin and violaxanthin, respectively. A balance of cellular zeaxanthin and violaxanthin levels is an important feature of NPQ and assists in the dissipation of stimulating light energy [15,19,20]. Additional photoprotection strategies have evolved in selected organisms to cope with high light conditions, e.g., by synthesis of astaxanthin, which has a strong antioxidative potential. The responsible enzyme, the ß-carotene ketolase (BKT), catalyzes the conversion of zeaxanthin to astaxanthin and ß-carotene to canthaxanthin at high efficiency, and astaxanthin accumulation is reported to contribute to ROS scavenging and highlight protection [21,22]. *C. reinhardtii* does not synthesize astaxanthin in a vegetative state; however, expression of an evolutionarily silenced ß-carotene ketolase (*Cr*BKT [23]) was recently restored by an innovative gene design [24,25]. Systematic metabolic engineering identified present limitations in the activity of involved ß-carotene hydroxylase and phytoene synthase as rate-limiting steps for increased astaxanthin production in *C. reinhardtii* [26]. By application of high light intensities (3000 µmol photons/m^2^/s) in a high-cell-density cultivation [2], accumulation of up to 23.5 mg/L was achieved after 94 h cultivation [26].

A complex cellular carotenoid profile assists in light-harvesting as well as excitation-energy quenching [19]; however, a tailored carotenoid pathway can support engineered astaxanthin biosynthesis by redirecting flux from the competing α-carotene biosynthesis and would simplify extraction efforts due to reduced carotenoid byproducts [27]. However, it is essential to maintain light protection activity for robust growth and cellular fitness, even under elevated light conditions. The application of genome-editing technology is readily established in *C. reinhardtii* [28,29,30,31,32,33] and was recently applied as a CRISPR-Cas9 ribonucleoprotein (RNP) complex for engineering an increased zeaxanthin accumulation by a functional double knockout of LCYE and ZEP [27]. Zeaxanthin contents were increased by 60% and resulted in up to 5.2 mg/L (7.3 mg/g) with elevated purity; however, this mutant showed impaired non-photochemical quenching capacity partly compensated for by an enhanced cyclic electron flow in phototrophic conditions [34]. However, individual effects from an LCYE knockout have not been tested, yet.

In this study, we demonstrate the potential of a tailored pigment biosynthesis pathway in *C. reinhardtii* production strain UVM4. We provide a detailed characterization of the growth performance of mutants lacking LCYE and the α-carotene route of carotenoid biosynthesis and demonstrate the increased capacity for efficient astaxanthin bioproduction in this alga.

## 2. Results and Discussion

### 2.1. Cas9-Mediated Knockout of CrLCYE by Integration of Selection Marker aphVII

**Figure 1:**
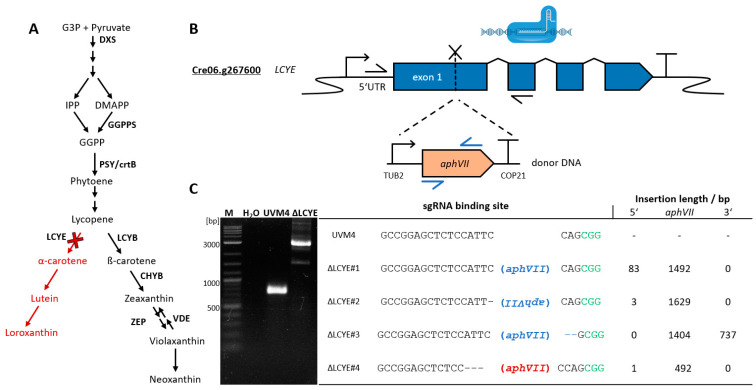
*C. reinhardtii* carotenoid pathway and targeted knockout of LCYE. (**A**) Simplified MEP and carotenoid biosynthesis pathway in *C. reinhardtii.* Relevant enzymes are depicted in bold. Red cross and red color display targeted knockout of LCYE and depleted α-carotene route in carotenoid synthesis. (**B**) Schematic illustration of RNP-mediated DNA double-strand break and integration of donor DNA containing an expression cassette composed of *C. reinhardtii* TUB2 promoter, *Streptomyces rimosus aphVII* CDS, and *C. reinhardtii* COP21 terminator. Arrows indicate oligonucleotide binding sites for amplification. Genetic constructs are illustrated using SBOL3.0 standard and genetic elements and are not at scale. (**C**) Exemplary agarose gel after separation of PCR products containing the LCYE locus from genomic DNA samples of parental cell line UVM4 and an exemplary ΔLCYE mutant. M—1 kb Plus DNA Ladder (NEB). The table presents the sgRNA binding sequence with the PAM motif in green and the respective sequences from four selected ΔLCYE mutants (ΔLCYE#1-4). Integration of *aphVII* is indicated in blue (functional ORF), inverted (integration in antisense direction), or red (partial integration). Length of integrated *aphVII* cassette and additional random DNA fragments at the 5′ and 3′ ends are indicated. G3P—glyceraldehyde 3-phosphate, DXS—1-deoxy-D-xylulose-5-phosphate synthase, IPP—isopentenyl pyrophosphate, DMAPP—dimethylallyl pyrophosphate, GGPP—geranylgeranyl pyrophosphate, GGPPS—geranylgeranyl pyrophosphate synthase, PSY(crtB)—phytoene synthase, LCYB—ß-lycopene cyclase, LCYE—ε-lycopene cyclase, CHYB—ß-carotene hydroxylase, BKT—ß-carotene ketolase, TUB2—ß-2-tubulin promoter, *aphVIII*—*Streptomyces rimosus* aminoglycoside 3′-phosphotransferase gene VII, COP21—*C. reinhardtii* chlamyopsin 2/1.

*C. reinhardtii* harbors a versatile carotenoid biosynthesis pathway divided into an α- and ß-carotene route resulting from competing ε/ß-lycopene cyclase activity (Figure 1A, LCYB and LCYE). The ß-carotene route provides essential precursors for recently revived astaxanthin production in *C. reinhardtii* [23], while LCYE activity towards α-carotene displays a substantial competing pathway (Figure 1A, red arrows). In the nuclear genome of *C. reinhardtii*, only a single gene locus for LCYE exists (gene ID: Cre06.g267600) and encodes for a 583 amino acid (aa) large protein, which has a predicted thylakoid luminal transfer peptide (at aa position 1-41, Pr: 0.4237 targetP 2.0 [35]). This gene was already described by engineered overexpression using genomic DNA amplification [14] and was subject of a double knockout in a ΔZEP background for increased zeaxanthin biosynthesis [27]; however, effects from an individual knockout have not been described, yet.

A suitable sgRNA binding site was previously predicted in the first exon (Figure 1B and C, position 185–208 bp past the start codon) using Cas-Designer [36]. RNP assembly was performed using in vitro synthesized sgRNAs and a commercial Cas9 protein followed by electroporation, as previously described [28]. Induced double-strand breaks at the target locus were used for non-homologous end joining (NHEJ)-based integration of a donor DNA element containing the *aphVII* resistance gene, including expression elements for transcription (Figure 1B, *C. reinhardtii* ß-2-tubulin promoter [37] and *C. reinhardtii* Chlamyopsin 2/1 terminator [38] based on pChlamy3 and used in a previous study [39]). Regenerated transformants exhibit resistance against the antibiotic hygromycin and were further characterized by PCR amplification (Figure 1B, black arrows).

In total, 96 transformants were isolated and further characterized via PCR amplification using oligonucleotides spanning the sgRNA target locus. No PCR product was observed in 28 of 96 transformants (29%), likely due to sequence alteration preventing amplification (e.g., genomic rearrangements, extended deletions, or insertions) mediated by NHEJ-based repair, the major mechanism of double-strand break repair events [40,41,42]. 64 of 96 transformants (67%) had similar product sizes compared to parental strain UVM4, indicating an unchanged LCYE locus (amplification length approx. 716 bp) and *aphVII* integration at random positions to confer resistance. However, it is possible that small insertions and deletions are present in some of these transformants that cannot be identified based on PCR product size, resulting in an underestimated editing efficiency. Undesired additional DNA integrations at random positions can cause off-target effects and phenotypical changes but may be removed by crossing using parental strains if required.

Four of 96 transformants (4%) showed larger PCR product sizes compared to the parental strain UVM4 (Figure 1C), indicating a potential integration of the *aphVII* cassette (length 1.631 bp) into the *LCYE* target site. The observed editing frequency is in line with recent findings for NHEJ-based editing [27,43] but lower compared to recent homology-directed repair strategies (up to 10–60% [28,30,32]).

Additional oligonucleotides were used to amplify junctions across the inserted DNA into the genomic LCYE locus (combinations of blue and black arrows, Figure 1B) followed by Sanger sequencing for sequence verification (Supplementary Figure S1). Mutants ΔLCYE#1 and 2 harbor an *aphVII* cassette with small deletions and few additional nucleotides of random origin at the 5′ site of the target locus (83 bp and 3 bp for ΔLCYE#1 and ΔLCYE#2, respectively). While the *aphVII* cassette was integrated into an antisense orientation in mutant ΔLCYE#2, the integration in ΔLCYE#4 was found to be only partial (492 bp) and likely non-functional with a second *aphVII* integration at a random position. Partial integration of donor DNA was previously described, using the same approach for LCYE targeting [27], and multiple insertions occur frequently during nuclear transformations of *C. reinhardtii* [44]. Mutant ΔLCYE#3 carries a full-length *aphVII* cassette in sense orientation followed by two short partial copies, with a short deletion (2 bp) at the 3′ junction of the target site. Error-free NHEJ integration without the loss of genomic DNA at the 3′ junction of the integration site was frequent in three of the four characterized transformants.

In the analyzed mutants, several junctions of ligated donor DNA ends show short (4 to 6 bp long) microhomologies to each other. In plants and algae, NHEJ is typically favored to repair DNA damage [40,41,42], and DNA ends can either be ligated directly or processed until terminal microhomologies stabilize DNA ends, a process called microhomology-mediated end joining (MMEJ) [45,46]. This could explain why especially fragmental and incomplete donor DNA integration occurred during genome editing. Despite the successful editing of LCYE and NHEJ-based integration of donor DNA, recent advantages in homologous directed repair (HDR) appear more favorable for precise genome editing and typically result in predictable DNA integrations [28,30,32]. We selected mutant ΔLCYE#3 for further characterization of biomass accumulation, endogenous pigment profiles, and engineered astaxanthin biosynthesis.

### 2.2. Growth Characterization and Pigment Profile of Mutant ΔLCYE#3

**Figure 2:**
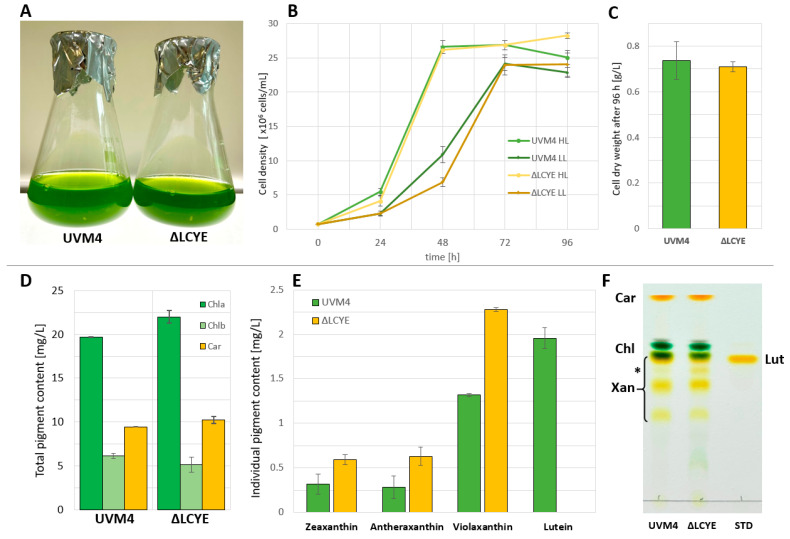
Growth performance of mutant ΔLCYE#3. (**A**) Culture of parental strain UVM4 and ΔLCYE#3 in a 100 mL shake flask 72 h past inoculation. (**B**) Cell-density measurements for UVM4 and ΔLCYE#3 during a cultivation period of 96 h in TAP medium and constant illumination of 100 µmol photons/m^2^/s (LL) and 500 µmol photons/m^2^/s (HL). (**C**) Gravimetric cell dry-weight quantification after 96 h cultivation in HL. (**D**) Total carotenoid content in TAP and HL after 72 h. (**E**) Pigment quantification via HPLC using acetone extracts from strain UVM4, ΔLCYE #3, and commercial standards. (**F**) Thin-layer chromatography of acetone extracts from strain UVM4, ΔLCYE #3, and a commercial lutein standard. Signals from α/ß-carotene (Car), chlorophyll a/b (Chl), xanthophylls (Xan), and lutein (Lut) are indicated at the respective positions. The asterisk indicates a strong change in signal patterns. All quantifications are given as mean values, and error bars display the standard deviation of three individual measurements from biological replicates.

Cultivation of parental strain UVM4 and mutant ΔLCYE#3 was performed in mixotrophic conditions using TAP medium [47] and continuous light at different intensities (100 (LL) and 500 (HL) μmol photons/m^2^/s). Cell densities and cell dry weights were recorded regularly throughout the course of cultivations until the stationary phase (Figure 2) was reached. Highlight application resulted in rapid growth during the first 48 h past inoculation; however, final cell densities after 96 h were comparable for both light conditions, which reached 28.3 × 10^6^ cells/mL for mutant ΔLCYE#3 compared to 25.1 × 10^6^ cells/mL for parental strain UVM4 (Figure 2B). The final cell dry weight was comparable for both strains and reached 0.73 and 0.71 g/L, respectively, indicating no phenotypical difference in growth and biomass generation for both strains. Exposure to high light intensities directs electron transfer towards the reduction of molecular oxygen, generating superoxide radicals (O_2_^−^.) at PSI [48] and, consequently, results in cellular damage. Excess excitation energy can be relieved by the lutein–loroxanthin cycle, which is absent in mutant ΔLCYE#3. However, this mutant was able to cope with selected conditions, likely due to compensatory NPQ mechanisms. Pulse-amplitude-modulation (PAM) fluorometry indicates comparable photosystem II quantum yields for both strains (UVM4: 0.595 ± 0.030; ΔLCYE#3 0.663 ± 0.021) and sufficient light tolerance.

Total carotenoid contents were quantified based on absorbance measurements of acetone extracts 72 h past inoculation and results indicate no change in the total amount of pigments (Figure 2D). HPLC and thin-layer chromatography were performed to characterize the endogenous pigment profile. In parental strain UVM4, cellular lutein contents were 2.27 mg/L, while lutein was completely absent in ΔLCYE#3, confirming the successful knockout of LCYE.

Our data suggest that knockout of *Cr*LCYE and the subsequent lack of the endogenous α-carotene route of carotenoid biosynthesis, including the lutein–loroxanthin cycle, does not limit cellular fitness compared to parental strain UVM4 under selected conditions. The carotenoid contents of the xanthophyll cycle were markedly increased, reflected by an elevated cellular content of zeaxanthin by 1.9 fold (from 0.31 mg/L in UVM4 to 0.59 mg/L in ΔLCYE#3), of antheraxanthin by 2.25 fold (from 0.28 to 0.63 mg/L), and violaxanthin by 1.8 fold (from 1.3 to 2.3 mg/L). These findings are in line with the TLC results (Figure 2F), where the signal intensity was reduced for a band at the running height of a commercial lutein standard and increased for intermediate carotenoid signals of the xanthophyll cycle (Figure 2F, asterisk). Likely, increased carbon channeling towards the ß-carotene route and a compensatory upregulation of xanthophyll biosynthesis to provide light protection are the main reasons for this increase under vegetative growth conditions.

### 2.3. Engineering Astaxanthin Biosynthesis in ΔLCYE#3

**Figure 3:**
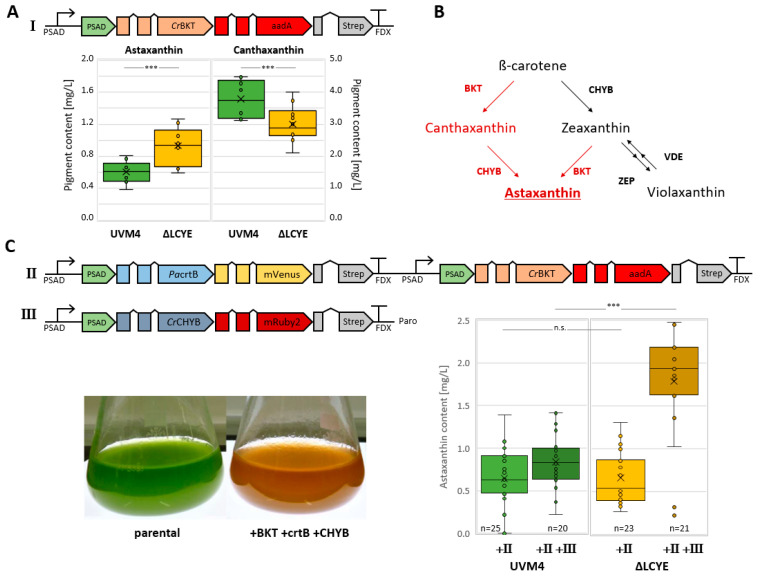
Engineering astaxanthin biosynthesis. (**A**) Schematic representation of genetic construct I for overexpression of *Cr*BKT as a fusion with selection marker *aadA* [26]. Astaxanthin and canthaxanthin contents for 10 selected transformants derived from parental strain UVM4 and ΔLCYE#3, respectively. The box and whisker plots indicate the distribution of astaxanthin production data from minimal (lowest line), lower quartile (bottom of box), median (central line), mean (cross), upper quartile (top of box), and maximal (top line) data points. Outliers are depicted as dots. Quantification was performed via HPLC UV/Vis detection (470 nm) from acetone extracts after 72 h mixotrophic cultivation in HL. (**B**) Astaxanthin and canthaxanthin biosynthesis in *C. reinhardtii* by expression of *Cr*BKT. (**C**) Schematic representation of genetic construct II and III for co-overexpression of *P. ananatis* crtB and *C. reinhardtii* CHYB [26]. Astaxanthin contents were quantified for selected transformants derived from parental strain UVM4 and ΔLCYE#3 in iterative transformations. Significance levels from an unpaired, two-sided Student’s *t*-test assuming non-homogenous variances are indicated (*** *p* < 0.01, n.s. *p* > 0.01). *Cr*BKT—*C. reinhardtii* ß-carotene ketolase, *Cr*CHYB—*C. reinhardtii* ß-carotene hydroxylase, ZEP—zeaxanthin epoxidase, VDE—violaxanthin de-epoxidase, PacrtB—*P. ananatis* phytoene synthase, mVenus —yellow fluorescence protein (YFP), mRuby2—red fluorescence protein (RFP), aadA—spectinomycin adenylyltransferase, PSAD—photosystem I reaction center subunit II, Strep—Strep-tagII epitope, FDX—*C. reinhardtii* ferredoxin 1 terminator.

Engineered overexpression of endogenous ß-carotene ketolase (*Cr*BKT) was enabled via optimized gene design [24,25,49] and revived evolutionary silenced astaxanthin biosynthesis in *C. reinhardtii*. Strategic metabolic engineering recently identified phytoene synthase (PSY/crtB) and ß-carotene hydroxylase (CHYB) as rate-limiting enzymes for increased astaxanthin bioproduction [26]. Nuclear transformations of parental strain UVM4 and mutant ΔLCYE#3 were performed to test the capacity for astaxanthin biosynthesis using previously designed plasmids (Figure 3). Transformation efficiency and selection were comparable for both strains, indicating no reduced cellular fitness mediated by the knockout of *Cr*LCYE. Initial overexpression of *Cr*BKT resulted in 0.61 ± 0.14 mg/L astaxanthin and 3.8 ± 0.6 mg/L canthaxanthin for transformants derived from parental strain UVM4 (n = 10), while transformants from ΔLCYE#3 reached 0.88 ± 0.23 mg/L astaxanthin (+44% compared to UVM4) and 2.9 ± 0.6 mg/L canthaxanthin (−24%). Increased astaxanthin levels indicate an increased flux towards zeaxanthin in mutant ΔLCYE#3 which resulted in reduced canthaxanthin formation as a byproduct during astaxanthin biosynthesis.

Iterative transformations of parental strains UVM4 and ΔLCYE#3 were performed using genetic constructs II and III (Figure 3C). Combined overexpression of *Cr*BKT and *Pa*crtB (construct II) resulted in an average astaxanthin accumulation of 0.6 ± 0.3 mg/L in selected transformants for both background strains. These values are comparable to previous findings where subsequent expression of *Cr*BKT and *Pa*crtB increased flux toward total carotenoids and sufficient activity of *Cr*BKT (reflected by increased canthaxanthin biosynthesis); however, terminal hydroxylation towards astaxanthin was still limited [26]. Additional co-overexpression of *Cr*CHYB induced a notable reddish coloration of cultures and increased astaxanthin accumulation to 0.8 ± 0.3 mg/L in UVM4 (Figure 3C II + III) or 1.8 ± 0.6 mg/L in mutant ΔLCYE#3 (II + III), respectively. Astaxanthin biosynthesis correlated with *Cr*CHYB expression levels based on fluorescence measurements and resulted in up to 2.44 mg/L after 72 h of mixotrophic cultivation. Biomass accumulation during astaxanthin production was comparable for both background strains (UVM4 and ΔLCYE#3) and derived transformants based on cell-density measurements and gravimetric quantifications (Supplementary Figure S2); however, cell densities were slightly increased for both engineered strains compared to their respective parental cell line.

Our results demonstrate that genome editing technology can successfully be used for the design of tailored carotenoid biosynthesis in *C. reinhardtii*, which supports astaxanthin production and would simplify downstream extraction strategies due to reduced pigment profile complexity. Astaxanthin accumulation was likely increased by substrate channeling towards the ß-carotene route of the endogenous carotenoid pathway and native upregulation of xanthophyll biosynthesis. Functional knockout of *Cr*LCYE and, consequently, lack of a lutein–loroxanthin cycle is not affecting the cellular fitness of *C. reinhardtii* UVM4 and depicts a valuable target for engineering. In a previous study, the introduction of an additional knockout of ZEP resulted in reduced chlorophyll contents, impaired growth, and non-photochemical quenching [27,34]. Further engineering is required to streamline carotenoid and astaxanthin biosynthesis in *C. reinhardtii*. Coupled with innovative overexpression strategies, these efforts can support the establishment of valuable bioproduction concepts using *C. reinhardtii* as a green cell factory.

## 3. Materials and Methods

### 3.1. Construct Design and Molecular Cloning

Coding sequences of *C. reinhardtii* BKT (UniProt: Q4VKB4), *Pantoea ananatis* phytoene synthase (crtB, UniProt: D4GFK9), and *C. reinhardtii* ß-carotene 3-hydroxylase (CHYB, UniProt: Q4VKB5) were previously designed as intron-containing algal transgenes [24,25,26] using Intronserter [49] and chemically synthesized (GenScript Biotech Corporation, Piscataway, NJ, USA) or PCR amplified via polymerase chain reaction performed using Q5 polymerase (New England Biolabs, Ipswich, MA, USA) according to the manufacturer’s protocol. All expression elements were assembled using the pOptimized vector system (Optimus Technologies, Pittsburgh, PA, USA) [23,25,26,50]. Transcription was driven by endogenous PSAD promoter [51] and FDX1 terminator [52,53]. The endogenous PSAD chloroplast-targeting peptide was employed to induce post-translational transport [51,53]. Cloning was performed using respective restriction enzyme digestion and ligation (T4 Ligase, New England Biolabs, Ipswich, MA, USA) according to the manufacturer’s protocol. Assembled DNA was used for the heat-shock transformation of chemically competent *E. coli* DH5a cells followed by selection on an LB medium containing respective antibiotics (300 mg L^−1^ ampicillin). Plasmid isolation was performed from overnight cultures using the peqGOLD Plasmid Miniprep Kit I (VWR International, Radnor, PA, USA) according to the manufacturer’s protocol.

### 3.2. Cultivation and Nuclear Transformation of C. reinhardtii

*Chlamydomonas reinhardtii* strain UVM4 [54] was routinely maintained on solid TRIS acetate–phosphate (TAP) medium [47] and cultivated in liquid TAP medium in microtiter plates or Erlenmeyer flasks at room temperature and continuous light (200 to 400 μmol photons m^−2^ s^−^^1^). Cell densities were quantified using a Z2 Particle Counter (Beckman Coulter Life Sciences, Brea, CA, USA) or a Countess II FL Automated Cell Counter (Thermo Fisher Scientific Inc., Waltham, MA, USA). The cell dry weight (CDW) was gravimetrically determined from 5–10 mL of culture pellets after centrifugation at 3000× *g* for 5 min and drying at 105 °C. Optical density was measured via absorbance quantification at 750 nm using a Genesys UV/Vis spectrophotometer (Thermo Fisher Scientific Inc., Waltham, MA, USA). Nuclear transformation was performed using 10 µg linearized plasmid DNA and glass bead agitation [55] followed by regeneration overnight at very low light intensities (5 μmol photons m^−2^ s^−1^). The selection was performed on a solid TAP medium containing appropriate antibiotics (10 mg L^−1^ paromomycin, 10 mg L^−1^ hygromycin, 200 mg L^−1^ spectinomycin, and 2.5 mg L^−1^ nourseothricin). For each construct, 288 transformants were isolated randomly, and the best 20 expressing transformants were identified using fluorescence measurements in a plant imaging system (NightShade LB 985, Berthold Technologies GmbH & Co. KG, Bad Wildbad, Germany) with appropriate filter sets for mVenus (excitation: 504 nm, emission: 530 nm) and mRuby2 (excitation: 560 nm, emission: 600 nm). Transformants were individually cultivated in microtiter plates and pooled samples were used for pigment quantification. The selection of strong expressing transformants for iterative transformations was performed using fluorescence microscopy (Leica MZ FLIII, Leica Microsystems GmbH, Wetzlar, Germany) with appropriate filter sets for mVenus (excitation: 510/20 nm, emission: 560/40 nm) and mRuby2 (excitation: 545/30 nm, emission: 620/60 nm). Pulse-amplitude-modulation (PAM) fluorometry was quantified using a Mini-Pam-II (Heinz Walz GmbH, Effeltrich, Germany).

### 3.3. CRISPR-Mediated Genome Editing and DNA Integration

Suitable sgRNA binding sites were previously identified [27] using the online tool Cas-Designer (BioTools, Inc., Jupiter, Fl, USA) at standard settings (CRISPR RGENTools, *Sp*Cas9, 5′-NGG-3′, *Chlamydomonas reinhardtii* v5.0, 20 bp crRNA length [36]). The GeneArt™ Precision sgRNA synthesis kit (Invitrogen, Thermo Fisher Scientific Inc., Waltham, MA, USA) was used according to the manufacturer’s protocol for sgRNA synthesis followed by purification using RNA Clean and Concentrator (Zymo Research Corporation, Orange, CA, USA). Functional RNPs were assembled by mixing 7 µg sgRNA with 8 µg *Sp*Cas9 protein (TrueCut Cas9 Protein v2, Invitrogen, Thermo Fisher Scientific Inc., Waltham, MA, USA) and incubated at room temperature for 15 min. Donor DNA was amplified from the *aphVII* cassette in a previously designed vector (pChlamy3 [27,33,56]) using the KOD One^TM^ PCR Master Mix (Toyobo Co., LTD., Osaka, Japan) and target specific oligonucleotides (forward 5′-GAATGTCTTTCTTGCGCTATGACACTTC-3′; reverse 5′-CAAGTACCATCAACTGACGTTACATTCTG-3′) followed by extraction from agarose gel (Dokdo-Prep^TM^, Gel extraction Kit, Elpis Biotech, Daejeon, Republic of Korea).

A UVM4 culture was routinely diluted and maintained in the early logarithmic phase (>1 × 10^6^ cells mL^−1^) prior to cultivation under nitrogen limitation for 24 h [28]. In total, 7 × 10^7^ cells were harvested, resuspended in TAP-sucrose (40 mM), and subjected to heat shock (40 °C, 20 min). Prior to electroporation (Gene Pulser Xcel System (Bio-Rad Laboratories, Inc., Hercules, CA, USA), square-wave protocol, 2 mm electrode-gap cuvettes, single pulse, 8 ms, and 250 V), the assembled RNPs and 1 µg donor DNA were added, mixed, and transferred to a sterile electroporation cuvette. Cells were regenerated for 10 min at room temperature prior to transfer to fresh TAP-sucrose medium for recovery at 5 µmol photons m^−2^ s^−1^. After 24 h, selection was applied by transfer on TAP agar plates containing appropriate antibiotics (10 mg L^−1^ hygromycin) for at least 5 days at 250–350 µmol photons m^−2^ s^−1^.

Genomic DNA from derived transformants was characterized by colony PCR [57] via amplification of the targeting locus (KOD One^TM^ PCR Master Mix (Toyobo Co., LTD., Osaka, Japan) or Q5 polymerase (New England Biolabs, Ipswich, MA, USA)) and accordingly from integrated DNA (Figure 1). PCR products were separated in 2% (*w*/*v*) agarose gels at 100 V for 30 min, isolated using a peqGold gel-extraction kit (VWR International, Radnor, PA, USA) and sequence identity was confirmed via sanger sequencing (Sequencing Core Facility, CeBiTec, Bielefeld University, Bielefeld, Germany).

### 3.4. Pigment Characterisation via Absorbance and Chromatography

Pigment characterization was performed as previously described [26]. Briefly, absorbance spectra from 350 to 750 nm were recorded for acetone extracts from cell culture pellets in a NanoDrop One photometer (Thermo Fisher Scientific Inc., Waltham, MA, USA), and total pigments were determined as previously described [26]. Thin-layer chromatography was performed with concentrated acetone extracts from cell pellets and separated on silica gel plates (Nano-ADAMANT 0.2 mm, Macherey and Nagel, Düren, Germany) using an appropriate running buffer (89.5% (*v*/*v*) petroleum, 10% (*v*/*v*) isopropanol, and 0.5% (*v*/*v*) water).

High-performance liquid chromatography (HPLC) was performed for quantification of endogenous pigments (Figure 2E) using a Shimadzu Prominence HPLC model LC-20AD (Shimadzu, Kyoto, Japan) equipped with a Spherisorb 5.0 μm ODS1 4.6 × 250 mm cartridge column (Waters Corporation, Milford, CT, USA), as previously described [27]. Astaxanthin and Canthaxanthin contents (Figure 3) were quantified using a Dionex UltiMate 3000 HPLC System (Thermo Fisher Scientific Inc., Waltham, MA, USA) and an Eurospher II 100-2 C18 column (100 mm × 2 mm, Knauer Wissenschaftliche Geräte GmbH, Berlin, Germany) was used with a diode array detector measuring at a wavelength of 470 nm. Carotenoids were separated using a gradient between 9:1 methanol–water (A) and methanol (B) at a flow rate of 1.0 mL min^−1^: 0 min B: 0%, 10 min B: up to 100%, 65 min B: 100%. Identification and quantification of chromatography signals were compared to commercially available authentic standards. Statistical evaluation has been performed using an unpaired, two-sided Student’s *t*-test assuming non-homogenous variances (significance levels are indicated as *** for *p* < 0.01 or n.s. for *p* > 0.01).

## Data Availability

All data generated in this study are available in this published article and Supplementary Materials.

## Supplementary Materials

The following supporting information can be downloaded at: https://www.mdpi.com/article/10.3390/plants13101393/s1, Figure S1: Sequence information of LCYE target site and donor DNA integration in four selected transformants. Figure S2: Biomass accumulation for parental strains and engineered production lines for efficient astaxanthin biosynthesis.

## References

[1] Freudenberg R.A., Wittemeier L., Einhaus A., Baier T., Kruse O. (2022). Advanced Pathway Engineering for Phototrophic Putrescine Production. Plant Biotechnol. J..

[2] Freudenberg R.A., Baier T., Einhaus A., Wobbe L., Kruse O. (2021). High Cell Density Cultivation Enables Efficient and Sustainable Recombinant Polyamine Production in the Microalga *Chlamydomonas reinhardtii*. Bioresour. Technol..

[3] Siitonen V., Probst A., Tóth G., Kourist R., Schroda M., Kosourov S., Allahverdiyeva Y. (2023). Engineered Green Alga *Chlamydomonas reinhardtii* as a Whole-Cell Photosynthetic Biocatalyst for Stepwise Photoproduction of H 2 and ε-Caprolactone. Green Chem..

[4] Griesbeck C., Kobl I., Heitzer M. (2006). *Chlamydomonas reinhardtii*: A Protein Expression System for Pharmaceutical and Biotechnological Proteins. Mol. Biotechnol..

[5] Yahya R.Z., Wellman G.B., Overmans S., Lauersen K.J. (2023). Engineered Production of Isoprene from the Model Green Microalga *Chlamydomonas reinhardtii*. Metab. Eng. Commun..

[6] Sreenikethanam A., Raj S., J R.B., Gugulothu P., Bajhaiya A.K. (2022). Genetic Engineering of Microalgae for Secondary Metabolite Production: Recent Developments, Challenges, and Future Prospects. Front. Bioeng. Biotechnol..

[7] Cao K., Cui Y., Sun F., Zhang H., Fan J., Ge B., Cao Y., Wang X., Zhu X., Wei Z. (2023). Metabolic Engineering and Synthetic Biology Strategies for Producing High-Value Natural Pigments in Microalgae. Biotechnol. Adv..

[8] Einhaus A., Baier T., Kruse O. (2023). Molecular Design of Microalgae as Sustainable Cell Factories. Trends Biotechnol..

[9] Cordero B.F., Couso I., León R., Rodríguez H., Vargas M.Á. (2011). Enhancement of Carotenoids Biosynthesis in *Chlamydomonas reinhardtii* by Nuclear Transformation Using a Phytoene Synthase Gene Isolated from Chlorella Zofingiensis. Appl. Microbiol. Biotechnol..

[10] van den Berg T.E., Croce R. (2022). The Loroxanthin Cycle: A New Type of Xanthophyll Cycle in Green Algae (Chlorophyta). Front. Plant Sci..

[11] McQuillan J.L., Cutolo E.A., Evans C., Pandhal J. (2023). Proteomic Characterization of a Lutein-Hyperaccumulating *Chlamydomonas reinhardtii* Mutant Reveals Photoprotection-Related Factors as Targets for Increasing Cellular Carotenoid Content. Biotechnol. Biofuels Bioprod..

[12] Rathod J.P., Vira C., Lali A.M., Prakash G. (2020). Metabolic Engineering of *Chlamydomonas reinhardtii* for Enhanced β-Carotene and Lutein Production. Appl. Biochem. Biotechnol..

[13] Ma R., Zhao X., Xie Y., Ho S.-H., Chen J. (2019). Enhancing Lutein Productivity of *Chlamydomonas* Sp. via High-Intensity Light Exposure with Corresponding Carotenogenic Genes Expression Profiles. Bioresour. Technol..

[14] Tokunaga S., Morimoto D., Koyama T., Kubo Y., Shiroi M., Ohara K., Higashine T., Mori Y., Nakagawa S., Sawayama S. (2021). Enhanced Lutein Production in *Chlamydomonas reinhardtii* by Overexpression of the Lycopene Epsilon Cyclase Gene. Appl. Biochem. Biotechnol..

[15] Grossman A.R., Lohr M., Im C.S. (2004). *Chlamydomonas reinhardtii* in the Landscape of Pigments. Annu. Rev. Genet..

[16] Esteban R., Matsubara S., Jiménez M.S., Morales D., Brito P., Lorenzo R., Fernández-Marín B., Becerril J.M., García-Plazaola J.I. (2010). Operation and Regulation of the Lutein Epoxide Cycle in Seedlings of Ocotea Foetens. Funct. Plant Biol..

[17] Matsubara S., Chen Y.-C., Caliandro R., Govindjee, Clegg R.M. (2011). Photosystem II Fluorescence Lifetime Imaging in Avocado Leaves: Contributions of the Lutein-Epoxide and Violaxanthin Cycles to Fluorescence Quenching. J. Photochem. Photobiol. B Biol..

[18] Leonelli L., Brooks M.D., Niyogi K.K. (2017). Engineering the Lutein Epoxide Cycle into Arabidopsis Thaliana. Proc. Natl. Acad. Sci. USA.

[19] Erickson E., Wakao S., Niyogi K.K. (2015). Light Stress and Photoprotection in *Chlamydomonas reinhardtii*. Plant J..

[20] Minagawa J., Tokutsu R. (2015). Dynamic Regulation of Photosynthesis in *Chlamydomonas reinhardtii*. Plant J..

[21] Cazzaniga S., Perozeni F., Baier T., Ballottari M. (2022). Engineering Astaxanthin Accumulation Reduces Photoinhibition and Increases Biomass Productivity under High Light in *Chlamydomonas reinhardtii*. Biotechnol. Biofuels Bioprod..

[22] Wang B., Zarka A., Trebst A., Boussiba S. (2003). Astaxanthin accumulation in haematococcus pluvialis (chlorophyceae) as an active photoprotective process under high irradiance 1. J. Phycol..

[23] Perozeni F., Cazzaniga S., Baier T., Zanoni F., Zoccatelli G., Lauersen K.J., Wobbe L., Ballottari M. (2020). Turning a Green Alga Red: Engineering Astaxanthin Biosynthesis by Intragenic Pseudogene Revival in *Chlamydomonas reinhardtii*. Plant Biotechnol. J..

[24] Baier T., Wichmann J., Kruse O., Lauersen K.J. (2018). Intron-Containing Algal Transgenes Mediate Efficient Recombinant Gene Expression in the Green Microalga *Chlamydomonas reinhardtii*. Nucleic Acids Res..

[25] Baier T., Jacobebbinghaus N., Einhaus A., Lauersen K.J., Kruse O. (2020). Introns Mediate Post-Transcriptional Enhancement of Nuclear Gene Expression in the Green Microalga *Chlamydomonas reinhardtii*. PLOS Genet..

[26] Amendola S., Kneip J.S., Meyer F., Perozeni F., Cazzaniga S., Lauersen K.J., Ballottari M., Baier T. (2023). Metabolic Engineering for Efficient Ketocarotenoid Accumulation in the Green Microalga *Chlamydomonas reinhardtii*. ACS Synth. Biol..

[27] Song I., Kim J., Baek K., Choi Y., Shin B., Jin E. (2020). The Generation of Metabolic Changes for the Production of High-Purity Zeaxanthin Mediated by CRISPR-Cas9 in *Chlamydomonas reinhardtii*. Microb. Cell Fact..

[28] Freudenberg R.A., Wittemeier L., Einhaus A., Baier T., Kruse O. (2022). The Spermidine Synthase Gene SPD1: A Novel Auxotrophic Marker for *Chlamydomonas reinhardtii* Designed by Enhanced CRISPR/Cas9 Gene Editing. Cells.

[29] Kelterborn S., Boehning F., Sizova I., Baidukova O., Evers H., Hegemann P. (2022). Gene Editing in Green Alga *Chlamydomonas reinhardtii* via CRISPR-Cas9 Ribonucleoproteins. Plant Synthetic Biology: Methods and Protocols.

[30] Angstenberger M., de Signori F., Vecchi V., Dall’Osto L., Bassi R. (2020). Cell Synchronization Enhances Nuclear Transformation and Genome Editing via Cas9 Enabling Homologous Recombination in *Chlamydomonas reinhardtii*. ACS Synth. Biol..

[31] Ferenczi A., Pyott D.E., Xipnitou A., Molnar A. (2017). Efficient Targeted DNA Editing and Replacement in *Chlamydomonas reinhardtii* Using Cpf1 Ribonucleoproteins and Single-Stranded DNA. Proc. Natl. Acad. Sci. USA.

[32] Nievergelt A.P., Diener D.R., Bogdanova A., Brown T., Pigino G. (2023). Efficient Precision Editing of Endogenous *Chlamydomonas reinhardtii* Genes with CRISPR-Cas. Cell Rep. Methods.

[33] Greiner A., Kelterborn S., Evers H., Kreimer G., Sizova I., Hegemann P. (2017). Targeting of Photoreceptor Genes in *Chlamydomonas reinhardtii* via Zinc-Finger Nucleases and CRISPR/Cas9. Plant Cell.

[34] Kim M., Cazzaniga S., Jang J., Pivato M., Kim G., Ballottari M., Jin E. (2024). Photoautotrophic Cultivation of a *Chlamydomonas reinhardtii* Mutant with Zeaxanthin as the Sole Xanthophyll. Biotechnol. Biofuels Bioprod..

[35] Almagro Armenteros J.J., Salvatore M., Emanuelsson O., Winther O., von Heijne G., Elofsson A., Nielsen H. (2019). Detecting Sequence Signals in Targeting Peptides Using Deep Learning. Life Sci. Alliance.

[36] Park J., Bae S., Kim J.-S. (2015). Cas-Designer: A Web-Based Tool for Choice of CRISPR-Cas9 Target Sites. Bioinformatics.

[37] Davies J.P., Weeks D.P., Grossman A.R. (1992). Expression of the Arylsulfatase Gene from the Beta 2-Tubulin Promoter in *Chlamydomonas reinhardtii*. Nucleic Acids Res..

[38] Lumbreras V., Stevens D.R., Purton S. (1998). Efficient Foreign Gene Expression in *Chlamydomonas reinhardtii* Mediated by an Endogenous Intron. Plant J..

[39] Kim J., Lee S., Baek K., Jin E. (2020). Site-Specific Gene Knock-Out and On-Site Heterologous Gene Overexpression in *Chlamydomonas reinhardtii* via a CRISPR-Cas9-Mediated Knock-in Method. Front. Plant Sci..

[40] Ferenczi A., Chew Y.P., Kroll E., von Koppenfels C., Hudson A., Molnar A. (2021). Mechanistic and Genetic Basis of Single-Strand Templated Repair at Cas12a-Induced DNA Breaks in *Chlamydomonas reinhardtii*. Nat. Commun..

[41] Plecenikova A., Mages W., Andrésson Ó.S., Hrossova D., Valuchova S., Vlcek D., Slaninova M. (2013). Studies on Recombination Processes in Two *Chlamydomonas reinhardtii* Endogenous Genes, NIT1 and ARG7. Protist.

[42] Puchta H. (2004). The Repair of Double-Strand Breaks in Plants: Mechanisms and Consequences for Genome Evolution. J. Exp. Bot..

[43] Cazzaniga S., Kim M., Pivato M., Perozeni F., Sardar S., D’Andrea C., Jin E., Ballottari M. (2023). Photosystem II Monomeric Antenna CP26 Plays a Key Role in Nonphotochemical Quenching in *Chlamydomonas*. Plant Physiol..

[44] Shahar N., Landman S., Weiner I., Elman T., Dafni E., Feldman Y., Tuller T., Yacoby I. (2020). The Integration of Multiple Nuclear-Encoded Transgenes in the Green Alga *Chlamydomonas reinhardtii* Results in Higher Transcription Levels. Front. Plant Sci..

[45] McVey M., Lee S.E. (2008). MMEJ Repair of Double-Strand Breaks (Director’s Cut): Deleted Sequences and Alternative Endings. Trends Genet..

[46] Pannunzio N.R., Watanabe G., Lieber M.R. (2018). Nonhomologous DNA End-Joining for Repair of DNA Double-Strand Breaks. J. Biol. Chem..

[47] Gorman D.S., Levine R.P. (1965). Cytochrome f and Plastocyanin: Their Sequence in the Photosynthetic Electron Transport Chain of *Chlamydomonas reinhardi*. Proc. Natl. Acad. Sci. USA.

[48] Triantaphylidès C., Krischke M., Hoeberichts F.A., Ksas B., Gresser G., Havaux M., Van Breusegem F., Mueller M.J. (2008). Singlet Oxygen Is the Major Reactive Oxygen Species Involved in Photooxidative Damage to Plants. Plant Physiol..

[49] Jaeger D., Baier T., Lauersen K.J. (2019). Intronserter, an Advanced Online Tool for Design of Intron Containing Transgenes. Algal Res..

[50] Lauersen K.J., Kruse O., Mussgnug J.H. (2015). Targeted Expression of Nuclear Transgenes in *Chlamydomonas reinhardtii* with a Versatile, Modular Vector Toolkit. Appl. Microbiol. Biotechnol..

[51] Fischer N., Rochaix J.D. (2001). The Flanking Regions of PsaD Drive Efficient Gene Expression in the Nucleus of the Green Alga *Chlamydomonas reinhardtii*. Mol. Genet. Genom..

[52] López-paz C., Liu D., Geng S., Umen J.G. (2018). Identification of *Chlamydomonas reinhardtii* Endogenous Genic Flanking Sequences for Improved Transgene Expression. Plant J..

[53] Einhaus A., Baier T., Rosenstengel M., Freudenberg R.A., Kruse O. (2021). Rational Promoter Engineering Enables Robust Terpene Production in Microalgae. ACS Synth. Biol..

[54] Neupert J., Karcher D., Bock R. (2009). Generation of *Chlamydomonas* Strains That Efficiently Express Nuclear Transgenes. Plant J..

[55] Kindle K.L. (1990). High Frequency Nuclear Transformation of *Chlamydomonas reinhardtii*. Proc. Natl. Acad. Sci. USA.

[56] Berthold P., Schmitt R., Mages W. (2002). An Engineered *Streptomyces Hygroscopicus* Aph 7″ Gene Mediates Dominant Resistance against Hygromycin B in *Chlamydomonas reinhardtii*. Protist.

[57] Cao M., Fu Y., Guo Y., Pan J. (2009). *Chlamydomonas* (*Chlorophyceae*) Colony PCR. Protoplasma.

