# Supplementary material for: CRISPR/Cas9-Mediated Knockout of the Lycopene ε-Cyclase for Efficient Astaxanthin Production in the Green Microalga Chlamydomonas reinhardtii
Source: Plants (Basel). 2024 May 17;13(10):1393. doi: 10.3390/plants13101393 (PMC11125023; doi:10.3390/plants13101393)
Supplement: Supplementary file 1 [file plants-13-01393-s001.zip › plants-2975455-supplementary.pdf]

LCYE sgRNA target site

		5′ insertion	*aphVII* cassette inserts / position and orientation	3′ insertion	
**UVM4**	GCCGGAGCTCTCCATTC	-	-	-	CAGCGG
**ΔLCYE#1**	GCCGGAGCTCTCCATTC	83	bp 26 – bp 1517	0	CAGCGG
**ΔLCYE#2**	GCCGGAGCTCTCCATT-	3	bp 1631 – bp 3	0	CAGCGG
**ΔLCYE#3**	GCCGGAGCTCTCCATTC	0	bp 53 – bp 1596; bp 1245 – bp 1589; bp 599 – 990	0	--GCGG
**ΔLCYE#4**	GCCGGAGCTCTCC----	1	bp 1175 – bp 1463; bp 1173 – bp 973	0	CAGCGG

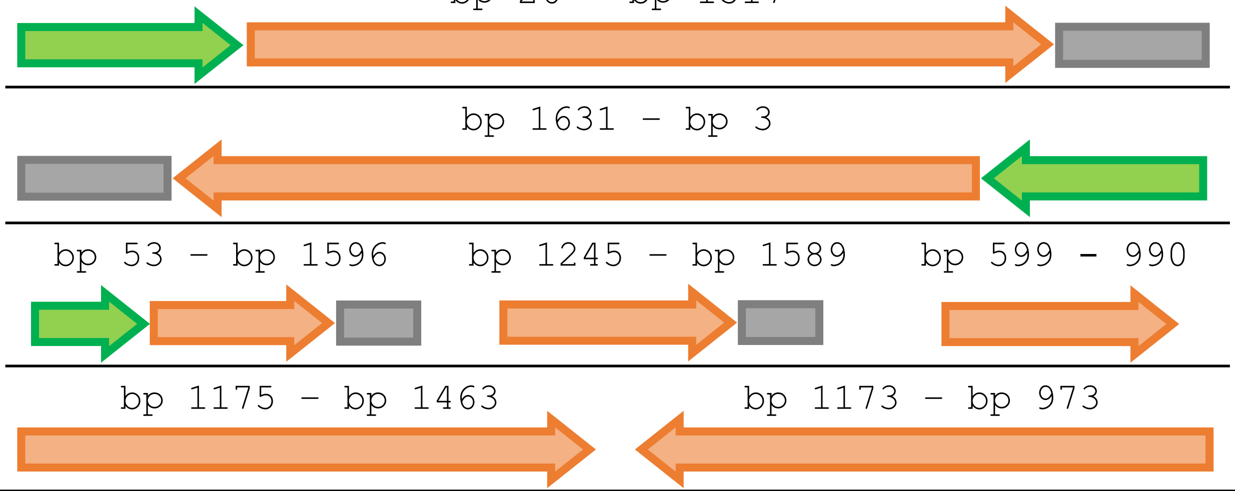

Supplementary Figure S1: The table presents the sgRNA binding sequence with PAM motif in red and the respective sequence for four selected ΔLCYE mutants (ΔLCYE#1-4). The position and orientation of individual *aphVII* cassette integrations is displayed for the promotor sequence (green arrow), *aphVII* coding sequence (orange arrow) and terminator (gray). Symbols do not represent actual sequence length. Length of integrated *aphVII* cassette and additional random DNA fragments at the 5′ and 3′ ends are indicated.

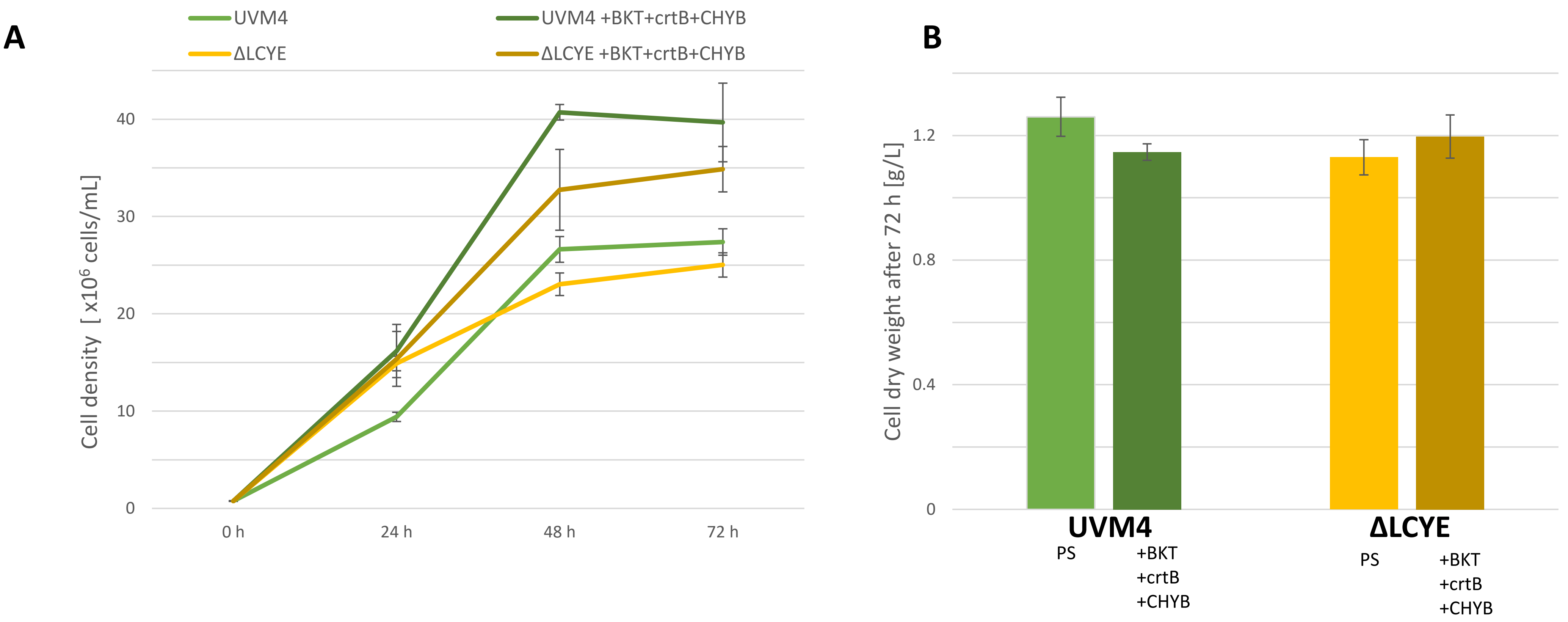

Supplementary Figure S2: Biomass accumulation for parental strains and engineered production lines for efficient astaxanthin biosynthesis. A) Cell density measurements for UVM4, ΔLCYE#3 and a selected transformant for each strain co-expressing *Cr*BKT, *Pa*crtB and *Cr*CHYB for production of astaxanthin (Figure 3). Cultivations were performed in 100 mL shake flasks for a cultivation period of 72 h in TAP medium and a constant illumination of 500 µmol photons/m^2/s. B) Gravimetric cell dry weight quantification after 72 h cultivation. All quantifications are given as mean values, and error bars display the standard deviation of three individual measurements from biological replicates. PS – parental strain
